# Mid-Term Results of a New Transobturator Cystocele Repair by Vaginal Patch Plastron without Mesh

**DOI:** 10.3390/jcm12144582

**Published:** 2023-07-10

**Authors:** Gautier Chene, Emanuele Cerruto, Stephanie Moret, Erdogan Nohuz

**Affiliations:** 1 Department of Gynecology, Hôpital Femme Mère Enfant (HFME), University hospital of Lyon, 59 Boulevard Pinel, 69500 Bron, France; 2 EMR 3738 CICLY, Claude Bernard Lyon 1 University, 69000 Lyon, France

**Keywords:** cystocele, vaginal plastron, pelvic organ prolapse, transobturator, pelvic floor dysfunction

## Abstract

Cystoceles are the most common prolapses. Limitation of the use of synthetic mesh has led to the comeback of native tissue repair procedures. We have developed a new transobturator technique with native tissue based on a mix of a vaginal plastron technique and the transobturator procedure. We present the functional and anatomical mid-term results. In this retrospective study, the vaginal plastron technique and the transobturator procedure were performed in 32 patients. Functional assessment with several validated quality-of-life questionnaires (SF-12, PFIQ-7, PFDI-20, PISQ12) and anatomical evaluation with pelvic examination were performed at 1, 6, and 12 months after surgery. The anatomical success rate was 94.4% at 12 months. There was one Clavien–Dindo grade 2 postoperative complication (one urinary tract infection). All of the quality-of-life scores were statistically significantly improved at one year follow-up. The transobturator technique combined with the vaginal plastron seems to be a promising, effective, innovative, and relevant technique for the repair of high-stage cystoceles.

## 1. Introduction

Since the recent ban on using transvaginal mesh for prolapse repair by the FDA [1], surgical techniques with native tissue are once again topical [2]. Concerning cystocele repair with native tissue, the conventional anterior colporrhaphy (based on several heterogeneous techniques of Halban’s fascia sutures) has been known for many years but may not be sufficient for severe prolapse with higher risk of recurrence [3]. Other autologous tissue techniques have been described:

- The vaginal patch plastron with six vaginal fixations elaborated by Crépin and Cosson is based on a vaginal strip still attached on the bladder with suspension to the tendinous arch of the pelvic fascia [4]. Short-term functional and anatomical results seem very good, with a success rate of 93% (44/47). However, the surgical technique may be difficult with severe complications (one peroperative hemorrhage, one ureteral section, one urethral injury) [4].
- Anterior sacrospinous fixation consisting of an anterior suspension to the sacrospinous ligament seems rather relevant, but mid- and long-term data are missing [5].

Recently, new transobturator techniques have been described [6,7,8] with promising mid-term results (objective cure rate of 90.5% (85/94) with only minor complications [8]).

We previously performed a transobturator cystocele technique with mesh with a long-term cure rate of 90% (over 150 procedures; data not published) [9].

We have developed a new transobturator technique with native tissue based on a mix of a vaginal plastron technique and the transobturator procedure. The advantages of vaginal plastron may combine bladder support and suspension with the transobturator fixation of the vaginal plastron. This technique may be suitable for the treatment of the median cystocele (thanks to the vaginal strip) as well as for the treatment of the paravaginal repair (with the bilateral suspension).

We propose a functional and anatomical mid-term retrospective series of this new transobturator technique with native tissue.

## 2. Materials and Methods

A single-center retrospective study of women with cystocele undergoing surgical repair by the transobturator technique with native tissue was conducted from September 2020 to March 2023 in accordance with the Declaration of Helsinki. This study was approved by the local Institutional Review Board (protocol code 22-5083 and date of approval: 21 December 2022) for studies involving humans. The ClinicalTrials.gov identifier was NCT05741567. All patients with symptomatic cystocele prolapse stage II or higher according to the Pelvic Organ Prolapse Quantification system (POP-Q) were invited to participate in the study. Consenting women agreed to provide baseline and follow-up data at 1, 6, and 12 months postoperatively on symptoms, anatomical correction, and quality of life, using specific questionnaires. If indicated, a concomitant posterior colporrhaphy with native tissue and/or a Richter’s sacrospinofixation could be performed. Vaginal hysterectomy was performed only in the case of symptomatic abnormalities confirmed via preoperative ultrasound (fibroids or adenomyosis, for example). No other mesh or sling (such as TVT or TOT) was used during the procedure. A preoperative urodynamic assessment was systematically performed.

### 2.1. Patient Reported Outcome Measures (PROMs)

The primary objective was to assess the degree of improvement after prolapse surgery using short- and mid-term health-related quality-of-life (HRQOL) questionnaires at 1, 6, and 12 months. We used the following validated quality-of-life questionnaires:

- The Patient Global Impression of Improvement (PGI-I) questionnaire for urogenital prolapse [10]. This is a self-administered, validated questionnaire that provides a global index of response to prolapse surgery. It is on a scale from 1 (very great improvement) to 7 (very great deterioration) that describes the current postoperative status compared to the preoperative status.
- The Short Form 12 (SF-12) questionnaire [11]. This is a self-administered, validated global quality-of-life questionnaire (score from 0 to 100) concerning the physical and mental components of quality of life. The higher the score, the better the quality of life.
- The Pelvic Floor Distress Inventory-20 (PFDI-20) questionnaire [12]. This is a self-administered, validated questionnaire for women with pelvic floor disorders. It measures the extent to which bowel, bladder, and pelvic symptoms bother the patient (from 0 to 100). The higher the score, the worse the quality of life.
- The Pelvic Floor Impact Questionnaire-7 (PFIQ-7) questionnaire [12]. This is a self-administered, validated questionnaire for women with pelvic floor disorders. It measures the extent to which bladder, bowel, or vaginal symptoms affect activities, relationships, and the emotional state of the patient (range 0–300). The higher the score, the worse the quality of life.
- The short form of the Pelvic Organ Prolapse/Urinary Incontinence Sexual Questionnaire (PISQ-12) [13]. This is a self-administered and validated instrument to evaluate the sexual function of women with pelvic organ prolapse. It measures three domains: behavioral–emotional, physical, and partner-related. Answers are graded on a 5-point Likert scale ranging from 1 to 4. Forty-eight is the maximum score; higher scores indicate better sexual function.
- Global pain was assessed using a 10 cm-VAS.

The secondary objective was objective anatomical success by clinical examination using the POP-Q classification. Cystocele stage II or higher (Ba > −1) was considered a failure of the surgical procedure.

### 2.2. Surgical Technique

A preoperative ultrasound was always performed to check for the presence or absence of uterine abnormalities. All procedures were performed under general anesthesia or locoregional anesthesia by an experienced urogynecologic surgeon (GC). The following surgical technical protocol was used, based on the technique described by Chabanon-Pouget et al. [9]:

Step 1: Incision of the vaginal plastron (diamond shape) after deep hydrodissection of the anterior vaginal wall. In order to avoid postoperative mucoceles, especially in non-menopausal women, the superficial epithelium from the plastron is destroyed by coagulation using a monopolar diathermy instrument or de-epidermized with a cold knife.

Step 2: Pubocervical fascia dissection and opening of both paravaginal spaces by blunt dissection towards the fascia of the obturator internus muscle.

**Figure 1:**
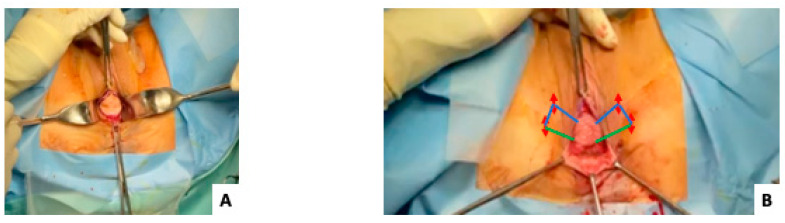
Surgical technique. (**A**) Incision of the vaginal plastron. (**B**) Two skin incisions (red arrows) are made closer to the internal part of the obturator foramen on both sides. The superior non-absorbable sutures (blue color) go to the superior skin incision and, afterwards, subcutaneously to the second skin incision. The inferior suture (green color on Figure 1) goes directly to the second skin incision.

Step 3: Two skin incisions (red arrows on Figure 1) are made closer to the internal part of the obturator foramen on both sides. The superior incision is at the level of the clitoris; the inferior one is 1.5 cm below and 1 cm lateral to the first stab skin incision.

Step 4: Two non-absorbable sutures (Prolene^®^ 2-0, Ethicon™, Johnson & Johnson, New Brunswick, NJ, USA) are fixed into the lateral part of the vaginal plastron.

**Figure 2:**
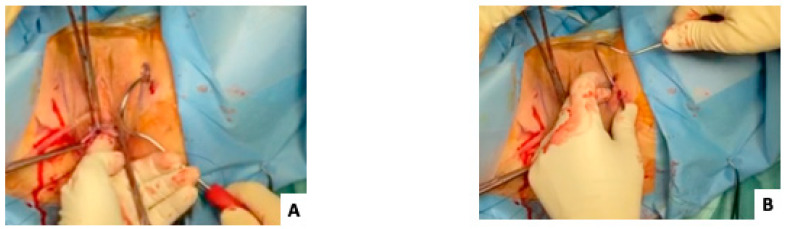
Surgical technique. (**A**,**B**) The outside-in tunneller is passed through the superior left skin incision and the obturator membrane towards the paravaginal space. The free end of the first suture is threaded through the open hole of the tunneller, which is removed by a reverse rotation. The tunneller is immediately reinserted subcutaneously through the same skin incision to the second inferior skin incision (technical trick described by Kalis et al. [2]).

Step 5: The superior non-absorbable sutures (blue color on Figure 1) go to the superior skin incision and, afterwards, subcutaneously to the second skin incision (technical trick described by Kalis et al. [2]). The outside-in tunneller (TOA5130 Tunneller^®^, AMI, Feldkirch, Austria) is passed through the superior left skin incision and the obturator membrane towards the paravaginal space. The free end of the first suture is threaded through the open hole of the tunneller, which is removed by a reverse rotation. The tunneller is immediately reinserted subcutaneously through the same skin incision to the second inferior skin incision (technical trick described by Kalis et al. [2]) (Figure 2).

Step 6: The inferior suture (green color on Figure 1) goes directly to the second skin incision.

Step 7: The outside-in tunneller is passed through the inferior left skin incision and the obturator membrane towards the paravaginal space (Figure 2). The free end of the second suture is threaded through the tunneller, which is removed.

**Figure 3:**
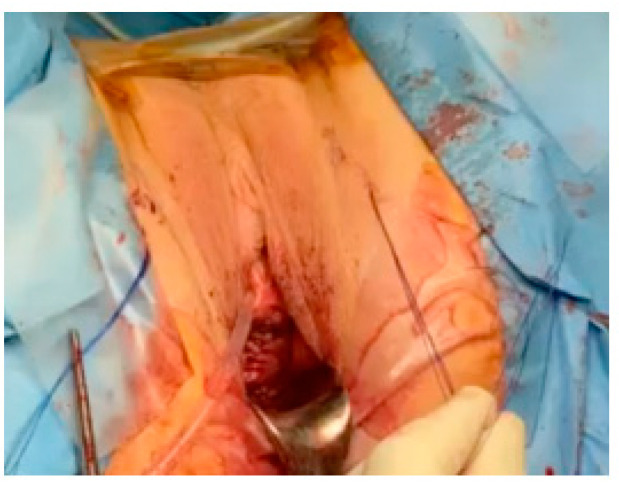
Final view. Tensioning process ensures correction of the cystocele by gentle traction on the sutures.

Step 8: The tensioning process ensures correction of the cystocele by gentle traction on the sutures. Both ends of the suture are tied and provided with a suitable anchor at the level of the aponeurotic muscle plane. The lateral fragments of the anterior vaginal wall cover the vaginal patch plastron at the end of the procedure. Skin incisions are closed (Figure 3).

An additional posterior colporrhaphy could be performed when indicated. The urinary catheter and vaginal gauze are placed and removed on the second postoperative day.

The duration of the operation and the perioperative and postoperative complications were also reported using the Clavien–Dindo classification [14], a validated scoring system for surgical complications.

### 2.3. Statistical Analysis

Statistical analysis was performed using the McNemar test and Student’s *t*-test for paired series. Data are described by their mean and standard deviation for continuous quantitative data and by their number and frequency for qualitative data. Statistical analysis was performed using SAS software (SAS Studio 3.6; SAS Institute Inc., Cary, CA, USA). A *p*-value less than 0.05 was considered significant.

## 3. Results

### 3.1. Surgical Outcomes

**Figure 4:**
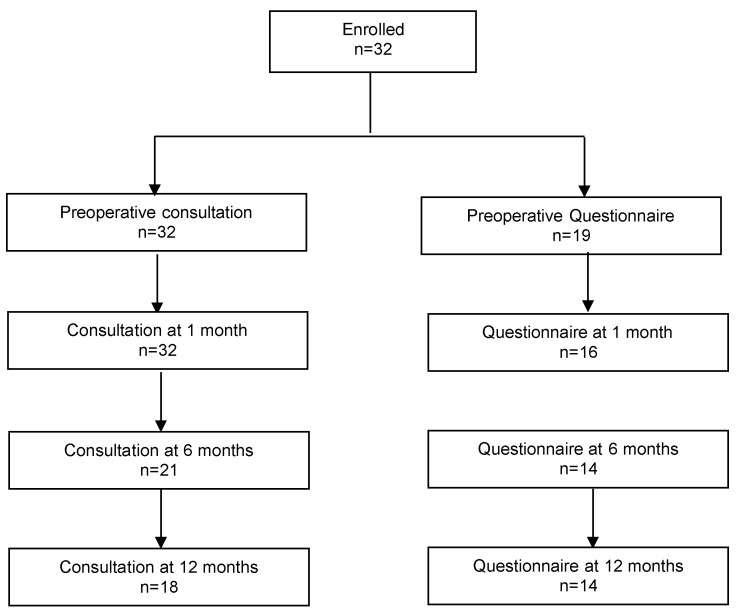
Study flowchart.

Thirty-two patients were included in this study (Figure 4). Patient characteristics and intraoperative data are presented in Table 1. The mean age was 63.5 years (±1.7). The majority of patients were postmenopausal. Most of them (31/32 = 96.8%) had a cystocele stage III-IV (point Ba > +1). The average duration of the surgical intervention was 70 ± 3 min. Concurrent posterior colporrhaphy was performed in nine patients (28.1%). Concomitant Richter’s sacrospinofixation was performed in six patients (18.7%). The estimated blood loss was 60 ± 23.2 mL. There were no perioperative or postoperative complications. Day one pain was moderate (2.1 ± 0.3 on VAS) and was related to the stab skin incisions. The duration of hospitalization was 2.5 days on average (Table 2).

### 3.2. Patient Outcomes

According to the POP-Q classification, the anatomical success rate concerning cystocele repair was 94.4% at 12 months (Table 3). One patient needed an anterior sacrospinofixation; three patients required bulking agent injections for stress urinary incontinence treatment. There was one Clavien–Dindo grade 2 postoperative complication: one case of urinary tract infection. There was a statistically non-significant decrease in the rate of SUI. Dysuria was significantly improved due to prolapse correction (Table 4).

According to the global index PGI-I, there was a significant improvement at 1, 6, and 12 months. The improvement in quality-of-life indicators was statistically significant at 12 months for SF-12, PFIQ-7, and PFDI-20. An assessment of the pain at the level of the operated area showed the virtual absence of pain. Recovery of sexuality was considered satisfactory in sexually active patients, with a significant improvement in the PISQ12 score at 12 months (Table 5).

## 4. Discussion

Anterior vaginal wall prolapse or cystoceles are the most common prolapses. The pathophysiology is complex, and cystoceles may be secondary to vaginal defects. These defects may be central or lateral (i.e., paravaginal defect) and often combined. Several surgical techniques have been described via the vaginal route: anterior colporrhaphy, vaginal wall flap, and paravaginal repair [2,3,4,5]. However, the relatively high recurrence rate has led to the use of synthetic mesh via the vaginal route. Sometimes severe complications (infection, vaginal erosion, mesh shrinkage, chronic pelvic pain, dyspareunia) have led to restrictions (or even a ban in some countries) on the use of mesh [1].

Therefore, there has been a return to autologous techniques. The transobturator route is well known to urogynecologists (especially with the TOT for SUI). Some transobturator techniques have recently been published. Kalis et al. [6] described a transobturator cystocele repair of level 2 paravaginal defect in a video article. Laufer et al. [7] published another video concerning another transobturator support approach. Last but not least, Sharifiaghdas [8] published the first prospective study of a transobturator approach using native vaginal wall tissue. She concluded that the mid-term results at 12 months were very promising, with an objective anatomical success rate of 90.5%.

The interest of the transobturator approach in combination with the vaginal patch is to repair the lateral defect (support with lateral sutures through the obturator foramen) on the one hand and, on the other hand, to repair the central defect (support with the vaginal patch). If indicated, a surgeon may add additional surgical procedures, such as vaginal hysterectomy [15], Richter’s sacrospinofixation [16], anterior sacrospinous vaginal vault suspension [5] (because uncorrected apical prolapse can increase the rate of recurrent anterior compartment prolapse), posterior colporrhaphy, etc.

In our study, functional and anatomic mid-term results were very good, with no peri-nor postoperative complications. The transobturator technique is quick and easy to learn. Moreover, it is possible that there may be a TOT effect of our technique, as the frequency of SUI decreased after the procedure.

We used the outside-in tunneller (TOA5130 Tunneller^®^, AMI, Feldkirch, Austria), which is a reusable instrument made of stainless steel for tunnelling during the treatment of female stress urinary incontinence with the suburethral slings. The Food and Drug Administration reclassified surgical instrumentation for use with urogynecologic surgical mesh from class I (general controls) to class II (special controls) and identified them as ‘‘specialized surgical instrumentation for use with urogynecologic surgical mesh’’ [17]. Special controls were necessary for the FDA to provide a reasonable assurance of safety and effectiveness of the device. In our opinion, this outside-in tunneller is a safe instrument and easy to use after a short learning curve (5 to 10 procedures). 

Limitations of the present study are the small number of patients, the absence of a comparative group, the lack of long-term follow-up, and patient drop-out in questionnaire filling (due to many quality-of-life questionnaires). The strengths lie in a new, easy, and promising surgical technique with relevant functional and anatomical long-term results. We chose several validated QoL questionnaires to assess all components of quality of life.

In a recent meta-analysis comparing native tissue repair versus transvaginal mesh interventions for the treatment of anterior vaginal prolapse, Capobianco et al. [18] showed that mesh repair surgery had higher anatomical cure and satisfaction rates but also higher post-surgical and late complications in comparison with native tissue technique. This suggests excellent safety outcomes from native tissue repair and the need to develop new effective surgical techniques without mesh.

Nevertheless, other prospective studies with larger cohorts are needed to confirm the interest of the transobturator approach as a relevant option for the correction of cystoceles.

## Figures and Tables

**Table 1:** Baseline characteristics of patients (*n* = 32).

Age	63.5 ± 1.7
BMI kg/m^2^, mean ± SD	26.3 ± 0.5
Parity	3.0 ± 0.3
Number of vaginal deliveries	2.8 ± 0.3
Menopause status, *n* (%)	27 (84.4)
Previous hysterectomy *n* (%)	7 (21.9)
Urinary stress incontinence *n* (%)	9 (28.1)
grade I	3
grade II	4
grade III	2
Urinary urgency *n* (%)	2 (6.2)
Dysuria *n* (%)	11 (34.4)
Preoperative UDA ^1^ *n* (%)	32 (100)
Mean closure pressure (cm H_2_O)	67.9 ± 6.7
Maximum flow (mL/s)	22.8 ± 2.1

Data are mean (± standard deviation) or *n* (%); ^1^ UDA: urodynamic assessment.

**Table 2:** Perioperative outcomes (*n* = 32).

Operative Time, min	70.0 ± 3.0
Concomitant vaginal hysterectomy	0 (0.0)
Concomitant posterior colporrhaphy	9 (28.1)
Concomitant Richter’s sacrospinofixation	6 (18.7)
Estimated blood loss ml	60.0 ± 23.2
Fever (≥38 °C)	0 (0.0)
Hemorrhage	0 (0.0)
Urinary infection	1 (3.1)
Pelvic infection	0 (0.0)
D-1 VAS pain scale	2.1 ± 0.3
Hospital stay (days)	2.5 ± 0.1

Data are mean (± standard deviation) or *n* (%).

**Table 3:** Anatomical results according to POP-Q classification.

	Preoperative *n* = 32	1 Month *n* = 32	6 Months *n* = 21	12 Months *n* = 18
Cystocele success		32 (100)	19 (90.5)	17 (94.4)
Cystocele	32 (100)	1 (3.1)	3 (14.3)	5 (27.8)
stage I	0	1	1	4
stage II	1	0	1	1
stage III	28	0	1	0
stage IV	3	0	0	0
Uterine prolapse success		32 (100)	20 (95.2)	18 (100)
Uterine prolapse	16 (50.0)	0 (0.0)	1 (7.8)	0 (0.0)
stage I	1	0	0	0
stage II	12	0	0	0
stage III	2	0	1	0
stage IV	1	0	0	0
Apical prolapse success		32 (100)	21 (100)	18 (100)
Apical prolapse	8 (25.0)	0 (0.0)	0 (0.0)	0 (0.0)
stage I	2	0	0	0
stage II	5	0	0	0
stage III	1	0	0	0
stage IV	0	0	0	0
Rectocele success		31 (96.9)	21 (100)	17 (94.4)
Rectocele	19 (59.4)	6 (18.7)	3 (14.3)	3 (16.7)
stage I	5	5	3	2
stage II	13	1	0	1
stage III	1	0	0	0
stage IV	0	0	0	0

Data are mean (± standard deviation) or *n* (%); success: stage 0 or I/non-success: stage II, III, IV.

**Table 4:** Follow-up.

	Preoperative *n* = 32	1 Month *n* = 32	*p* *	6 Months *n* = 21	*p* *
SUI ^a^	9 (28.1)	3 (9.4)	0.07	3 (14.3)	0.06
grade I	3	1		0	
grade II	4	2		3	
grade III	2	0		0	
Urinary urgency	2 (6.2)	0 (0.0)	-	0 (0.0)	-
Dysuria	11 (34.4)	1 (3.1)	0.006	1 (4.8)	0.06
Urinary infection		1 (3.2)		0 (0.0)	
Vaginal infection		0 (0.0)		0 (0.0)	
	**Preoperative** ***n* = 32**		**12 months** ***n* = 18**		*p* *
SUI ^a^	9 (28.1)		4 (22.2)		0.62
grade I	3		3		
grade II	4		1		
grade III	2		0		
Urinary urgency	2 (6.2)		1 (5.6)		1.00
Dysuria	11 (34.4)		0 (0.0)		-
Urinary infection			0 (0.0)		
Vaginal infection			0 (0.0)		

Data are mean (± standard deviation) or *n* (%); * *p*: compared to preoperative data; ^a^ stress urinary incontinence.

**Table 5:** Quality-of-life scores.

	Preoperative *n* = 19	1 Month *n* = 16	*p* *	6 Months *n* = 14	*p* *
PGI-I ^a^		1.7 ± 0.3		1.8 ± 0.3	
PGI-I ^a^ (scores 1,2,3)		14 (87.5)		13 (92.9)	
SF-12					
Physical score	43.7 ± 2.6	50.9 ±1.6	0.02	53.5 ± 1.7	0.02
Mental score	46.3 ± 2.7	55.9 ± 1.8	0.0006	57.2 ± 1.7	0.0006
PFIQ-7 ^b^	105.0 ± 18.5	2.7 ± 1.4	0.0001	2.0 ± 2.0	0.001
UIQ-7	44.3 ± 6.4	1.5 ± 0.9	<0.0001	0.0 ± 0.0	0.0007
CRAIQ-7	25.1 ± 6.5	0.6 ± 0.4	0.005	0.0 ± 0.0	0.02
POPIQ-7	35.6 ± 7.2	0.6 ± 0.4	0.0007	2.0 ± 2.0	0.01
PFDI-20 ^c^	139.7 ± 14.1	9.8 ± 2.7	<0.0001	9.5 ± 4.2	<0.0001
POPDI-6	65.3 ± 5.3	1.8 ± 0.8	<0.0001	4.2 ± 2.9	<0.0001
CRADI-8	18.9 ± 4.8	2.5 ± 1.1	0.007	2.2 ± 0.9	0.006
UDI-6	55.5 ± 6.6	5.5 ± 2.2	<0.0001	3.1 ± 1.3	0.0003
Global pain ^d^		0.8 ± 0.4		0.1 ± 0.1	
PISQ12 ^e^	30.3 ± 3.3	45.0	-	39.7 ± 1.5	0.10
	**Preoperative** ***n* = 19**		**12 Months** ***n* = 14**		*p* *
PGI-I ^a^			1.3 ± 0.2		
PGI-I ^a^ (scores 1,2,3)			14 (100)		
SF-12					
Physical score	43.7 ± 2.6		54.8 ± 1.2		0.002
Mental score	46.3 ± 2.7		54.3 ± 3.4		0.04
PFIQ-7 ^b^	105.0 ± 18.5		2.0 ±1.4		0.001
UIQ-7	44.3 ± 6.4		0.7 ± 0.5		0.0003
CRAIQ-7	25.1 ± 6.5		0.7 ± 0.5		0.02
POPIQ-7	35.6 ± 7.2		0.7 ± 0.5		0.002
PFDI-20 ^c^	139.7 ± 14.1		4.5 ± 3.2		<0.0001
POPDI-6	65.3 ± 5.3		0.6 ± 0.6		<0.0001
CRADI-8	18.9 ± 4.8		1.6 ± 1.1		0.002
UDI-6	55.5 ± 6.6		2.4 ± 1.5		<0.0001
Global pain ^d^			0.3 ± 0.3		
PISQ12 ^e^	30.3 ± 3.3		42.3 ± 1.5		0.04

Data are mean (± standard deviation) or *n* (%); ^a^ PGI-I: Patient Global Impressions scale—Improvement; ^b^ PFIQ: Pelvic Floor Impact Questionnaire; ^c^ PFDI: Pelvic Floor Distress Inventory; ^d^ global pain (10 cm VAS); ^e^ PISQ: Pelvic Organ Prolapse/Urinary Incontinence Sexual Questionnaire; * *p*: compared to preoperative data.

## Data Availability

The datasets used are available from the corresponding author on reasonable request.
